# The Role of Resveratrol in Liver Disease: A Comprehensive Review from In Vitro to Clinical Trials

**DOI:** 10.3390/nu13030933

**Published:** 2021-03-13

**Authors:** Carmine Izzo, Monica Annunziata, Giuseppe Melara, Roberta Sciorio, Marcello Dallio, Mario Masarone, Alessandro Federico, Marcello Persico

**Affiliations:** 1 Internal Medicine and Hepatology Division, Department of Medicine and Surgery “Scuola Medica Salernitana”, University of Salerno, Baronissi, 84081 Salerno, Italy; carmine.izzo93@gmail.com (C.I.); annunziata.monica@gmail.com (M.A.); gius.melara@gmail.com (G.M.); robertasciorio92@gmail.com (R.S.); mario.masarone@gmail.com (M.M.); 2 Department of Medicine, Surgery and Dentistry “Scuola Medica Salernitana”, University of Salerno, Baronissi, 84081 Salerno, Italy; 3 Hepatogastroenterology Division, Department of Precision Medicine, University of Campania Luigi Vanvitelli, Via Pansini 5, 80131 Naples, Italy; marcello.dallio@gmail.com (M.D.); alessandro.federico@unicampania.it (A.F.)

**Keywords:** resveratrol, non-alcoholic fatty liver disease, nutraceutical, oxidative stress

## Abstract

Many studies have shown that resveratrol has a lot of therapeutic effects on liver disorders. Its administration can significantly increase the survival rate after liver transplantation, reduce fat deposition and ischemia-induced necrosis and apoptosis in Wistar rats. Resveratrol can provide Liver protection against chemical, cholestatic, and alcohol-mediated damage. It can improve glucose metabolism and lipid profile, reduce liver fibrosis, and steatosis. Additionally, it is capable of altering the fatty acid composition of the liver cells. Resveratrol may be a potential treatment option for the management of non-alcoholic fatty liver disease (NAFLD) due to its anti-inflammatory, antioxidant, and calorie-restricting effects. There are also studies that have evaluated the effect of resveratrol on lipid and liver enzyme profiles among patients with metabolic syndrome (MetS) and related disorders. Based on the extent of liver disease worldwide and the need to find new treatment possibilities, this review critically examines current in vitro and in vivo preclinical studies and human clinical studies related to liver protection.

## 1. Introduction

Resveratrol (3,5,4′-trihydroxy-trans-stilbene) is a non-flavonoid phenol produced by numerous plants in response to bacteria or fungi colonization. It can be found mainly in grape peel, blueberries, raspberries, mulberries, and peanuts while its high concentration in red wine explains, in part, the relatively low incidence of cardiovascular disease in the French population despite the prevalence of a high-fat diet (HFD) among this population [1]. Numerous studies have shown that resveratrol can prevent or slow the progression of a wide variety of diseases, including malignant tumours, neurodegenerative diseases, cardiovascular disorders, ischemic lesions, and viral infections [2,3,4,5]. 

Interestingly one of the most important scientific fields in this context is currently represented by its potential therapeutic effects in liver diseases [6,7]. 

The mechanisms underlying the beneficial effects of resveratrol have not been fully elucidated and have mainly been related to its antioxidant activity [8]. This property has been shown to protect tissues such as the liver, kidneys, and brain from a variety of types of damage caused by oxidative stress [9]. Considering the spread of liver diseases—in particular, metabolic (dysfunction) associated fatty liver disease (MAFLD)—all over the world and the necessity for new useful therapeutic approaches, efforts made by the scientific community in exploring the role of nutraceuticals in this scenario are crucial for assessing the applicability of the evidence obtained from in vitro and clinical trials and translating it to routine clinical practice. For these reasons, in this comprehensive review, we analyzed the current scientific knowledge about the use of resveratrol for treatment of liver diseases from in vitro models up to clinical trials.

## 2. Resveratrol and Liver Disease: Molecular Mechanisms

### 2.1. Steatosis

Obesity is a major risk factor for non-alcoholic fatty liver disease [10]. Resveratrol possesses beneficial properties that are fundamental in preventing this metabolic condition, therefore attenuating liver inflammation [11,12,13,14]. The effects of resveratrol on non-alcoholic fatty liver disease (NAFLD) are correlated to the regulation of Low-Density Lipoprotein (LDL) receptors [15].

Poulsen et al. [11] showed that resveratrol reduces the diet-induced accumulation of hepatic fat through increased oxidation of fatty acids and reductions in lipogenesis. These effects are mediated by the activation of the AMPK/SIRT1 (AMP-activated protein kinase/sirtuin 1) axis [16,17]. RSV (resveratrol) supplementation in rodents fed with a high-fat diet demonstrated an increased number of mitochondria and, in particular, an increase in hepatic decoupling protein 2 expression, which could be involved in the normalization of the hepatic fat content [18]. Resveratrol significantly reduced TAG (triacylglycerols) and cholesterol, as well as the number and size of lipid droplets [19,20]. It would appear that a low dose of resveratrol (0.005%) is more effective than higher doses (0.02%) at reducing the development of hepatic steatosis, along with increases in body weight, plasma TAG levels, and total cholesterol levels [21]. The observed effects could be related to the suppression of fatty acid (FA) synthase, glucose-6-phosphate dehydrogenase, and phosphatide phosphohydrolase, whilst also partly being a consequence of the activation of FA oxidation in hepatic adipose tissue [22]. 

Bujanda et al. [23] investigated the action of resveratrol on hepatic steatosis in an animal model of a high carbohydrate-fat diet. They found that liver fat deposition was reduced in the resveratrol treated group (10 mg/day) compared to the controls. This effect was mainly related to an increase in SOD (superoxide dismutase), GPx (glutathione peroxidase), CAT (catalase), and a reduction in NOS (nitric oxide synthase) activity [23]. Focusing attention on the molecular mechanisms related to the role of resveratrol in alcoholic fatty liver disease—the activation of SIRT1 (sirtuin 1) and AMPK (AMP-activated kinase)—two critical signalling molecules that regulate the metabolic pathways of liver lipids were highlighted [24,25]. Resveratrol is a potent activator of both SIRT1 and AMPK [26]. Chronic consumption of ethanol causes an inhibition of the hepatic SIRT1-AMPK signalling system; resveratrol works by reversing this action and preventing the development of alcoholic fatty liver disease [27]. In fact, in ethanol-fed mice, RSV treatment has been shown to increase hepatic SIRT1 expression and stimulate AMPK activity, improving metabolic lipid homeostasis and antagonizing the development and progression of the disease [28].

### 2.2. Oxidative Stress and Inflammation 

Resveratrol has been shown to elicit relevant antioxidant properties in a wide range of hepatic disorders [29,30,31,32,33]. Specifically, its effect is mainly exerted by the reduction of RNS (ROS/reactive nitrogen species), elimination of direct free radicals, and an improvement in endogenous antioxidant enzyme activity (e.g., SOD, CAT, GSH) [34,35]. Moreover, RSV promotes the synthesis of antioxidant molecules and the expression of related genes involved in the biogenesis of mitochondrial energy, mainly through the AMPK/SIRT1/Nrf2 (nuclear factor 2), ERK/p38 (signal-regulated extracellular kinase), MAPK (mitogen-activated protein kinase), and PTEN/Akt (phosphatase and tensin homolog/protein kinase B) signalling pathways. In turn, this induces autophagy through an mTOR-dependent (target of rapamycin in mammals) or TFEB-dependent (EB transcription factor) pathway [36,37]. Erythroid nuclear transcription factor-2 (Nrf2; encoded by the NFE2L2 gene in humans) is a transcription factor that regulates the gene expression of a large variety of antioxidant and cytoprotective enzymes through a promoter sequence called ARE (antioxidant response element) [38,39]. In this sense, resveratrol acts as an antioxidant by inhibiting Nrf2 ubiquitination, safeguarding the maintenance of its crucial functioning [40]. Nrf2, in turn, translocates into the nucleus, inducing the transcription of various antioxidant genes, such as SOD and CAT improving, then the antioxidant defence [41]. 

Resveratrol promotes the transcriptional functions of FoxOs (forkhead box protein O1) in order to facilitate the transcription of various antioxidant genes such as HO-1 (heme oxygenase 1) [42]. Moreover, RSV up-regulates PTEN, an important PI3K (phosphatidylinositol 3-kinase) antagonist, by blocking the activation of Akt, leading to reduced phosphorylation of FoxOs, and in turn increasing its nuclear activity [43]. 

The protein kinase activated by AMP (AMPK), in conditions of energy depletion, plays an important role in the regulation of energy homeostasis, tumorigenesis, and longevity, in addition to an antioxidant role, in response to resveratrol [44]. In fact, AMPK directly phosphorylates human FoxO1 in vitro and increases FoxO1-dependent transcription of manganese superoxide dismutase and catalase [30].

### 2.3. Liver Fibrosis

Oxidative stress plays a crucial role, in the NAFLD setting, promoting progression towards liver fibrosis [45]. In this regard, RSV seems to play an interesting role in the regulation of de novo fibrogenesis deposition, acting on several key pathways [46]. In fact, RSV administration reduces portal pressures, the activation of hepatic stellate cells, and improves hepatic endothelial functioning in cirrhotic rats, with an overall positive effect on cirrhosis and portal hypertension [47,48,49]. Specifically, the administration of resveratrol (10 and 20 mg/kg/day) to cirrhotic rats for 2 weeks showed reductions in portal pressure without significant changes in systemic hemodynamics. This effect was attributed to improved acetylcholine vasodilatory responsiveness associated with reduced production of TXA2 (thromboxane A2) and, increased endothelial NO synthesis, which is, in turn, associated with a consequential significant reduction in hepatic fibrosis. Moreover, in this complex biologic network, the well-known action of RSV on the expression of collagen-1, TGF-β (transforming growth factor β), NF-κB (nuclear factor kappa-light-chain-enhancer of activated B cells), desmin mRNA, and α-SMA (α-smooth muscle actin) protein expression seems to play a crucial role in hepatocytes [47,50,51]. 

Another interesting study, using an animal DMN (dimethyl-nitrosamine) model of liver fibrosis, demonstrated the effect of RVS in reducing the liver tissue infiltration of inflammatory cells and fibrosis deposition [52]. The molecular mechanisms highlighted as underlying this process in cultured cells of hepatocytes included reduction in MDA (malondialdehyde) levels and increases in GPx and SOD levels, as well as an inhibition of mRNA expression of inflammatory mediators, including inducible NO, TNF-α (tumour necrosis factor-α), and IL-1β (interleukin-1β) [53,54]. 

### 2.4. Cholestatic Liver Damage

A cascade of inflammatory responses can lead to liver injury, through hepatic regeneration and fibrogenesis. Bile salts, associated with cholestasis, are involved in these processes. 

An interesting study evaluated the hepatoprotective effects of resveratrol against cholestatic damage in a rat model via bile duct ligation. Resveratrol significantly reduced TNF-α and IL-6 mRNA in cultured cells of hepatocytes, reducing the number of CD68 (+) Kupffer cells recruited in the liver. In the same experimental model, there was a reduction in fibrotic tissue deposition that was mainly linked to lower TIMP-1 (tissue inhibitor of metalloproteinase-1) and collagen Iα1 mRNA expression. These findings were associated with an increase in Ki67 (+) hepatocytes, thereby promoting their proliferation [55]. Another study, involving the application of a model of cholestatic liver damage induced by ANIT (α-naphthyl isothiocyanate), showed that resveratrol pre-treatment effectively attenuated ANIT-induced acute cholestasis and liver injury in rats [56,57]. In this setting, the suppression of neutrophil infiltration, and the upregulation of the expression of transporters and liver enzymes, were the mechanisms identified as being responsible for the reduction in bile acid accumulation and the related damage [58]. 

### 2.5. Hepatocellular Carcinoma and Metastasis

The anti-proliferative activity of resveratrol is mainly linked to triggering apoptosis of tumour cells [59,60,61,62,63,64]. Apoptosis can be activated through two main pathways: the intrinsic pathway mediated by mitochondria-apoptosomes and the extrinsic pathway induced by death receptors [65,66]. Activation of death receptors of the TNF (tumour necrosis factor) receptor superfamily, p. Eg, Fas (CD95/APO-1) or TRAIL (TNF-related apoptosis-inducing ligand receptors) causes the release of the caspase-8 initiator, which can mediate the apoptosis signal through direct cleavage of downstream caspase effectors, such as caspase-3 [67]. The intrinsic pathway is triggered by the dispensing of apoptogenic factors such as Omi/HtrA2 (serine protease HTRA2, mitochondrial), Smac/DIABLO (second mitochondria-derived activator of caspases/direct IAP (apoptic protein inhibitors) binding protein with low pI), cytochrome c, apoptosis-inducing factors (AIF), endonuclease G, caspase-2 and caspase-9 from the mitochondrial intermembrane space [68]. Dissemination of cytochrome c into the cytosol activates caspase-3 through the creation of an apoptosome complex containing cytochrome c/apoptotic protease activating factor-1 (Apaf-1)/caspase-9; Omi/HtrA2 and Smac/DIABLO encourage caspase activation by neutralizing the effects of apoptotic protein inhibitors (IAPs) [68,69]. Focusing on these mechanisms, in cultured cells of hepatocytes, resveratrol induces cancer cell death by modulating various transduction pathways through the regulation of Fas and Fas-ligand (FasL) levels [70,71]. Moreover, the ability of RSV to induce apoptosis by inhibiting the PI3K/Akt/mTOR pathway has been demostrated [72,73,74,75,76,77], thus, modulating the mitogen-activated protein kinase (MAPK) cascade [75,76,78] and inhibiting the activation of NF-κB [79,80]. In addition, RSV’s anticancer properties seem to be not only related to its biologic influence on the apoptosis cycle, as highlighted by the inhibition of signal transducers and activators of transcription 3 (STAT3) [81], a key element in tumorigenesis due to its induction of tumour cell proliferation, survival, invasion, angiogenesis, and metastasis [82,83]. A reduction in both HCC (hepatocellular carcinoma) occurrence and the number of hepatic nodules represent the basis of RSV’s protective mechanisms, as highlighted in animal models using carcinogenic chemicals such as DENA (diethyl nitrosamine) [84], DENA plus phenobarbital [62,85], and DENA plus 2-AAF (2-acetylaminofluorene) [86] or transgenic mice (e.g., transgenic mice expressing the hepatitis B viral protein (HBx)) [87]. Dietary resveratrol restored cellular antioxidant defences, thus, reducing DENA-induced lipid peroxidation and increasing protein carbonyl formation. It also increased hepatic Nrf2 and reduced iNOS (inducible nitric oxide synthase) expression. Moreover, though its activity on Nrf2, in another experimental context, a direct anti-inflammatory effect linked to a reduction in the levels and expressions of hepatic TNF-α, IL-1β, and IL-6 was highlighted [88]. Furthermore, resveratrol has been shown to elicit a remarkable anti-angiogenic effect during the development of DENA-induced hepatocellular carcinogenesis, blocking VEGF (vascular endothelial growth factor) expression through HIF-1α (hypoxia-inducible factor-1α) downregulation [89]. It has also been demonstrated that resveratrol is involved in the inhibition of cell cycle progression through reductions in the levels of p34cdc2 and cyclin B1 [90]. An initial study in 2001, reported that RSV at doses of 100 and 200 µM inhibited hepatoma cell proliferation and suppressed hepatoma cell invasion at concentrations of 25 µM [91]. Polyphenol suppressed the expression of antioxidant proteins that protect cells from ROS or cellular oxidative damage in SK-HEP-1 cells. This occurs by repressing the expression of these proteins and, thus, resveratrol is involved in increasing the susceptibility of cancer cells to oxidative stress. Paradoxically, the molecular mechanism underlying the anti-tumour role of resveratrol is its pro-oxidant action. Furthermore, in human HepG2 cells, the anti-tumour effects of RSV were attributed to its capacity to mediate cell cycle arrest in the G1 and G2/M phases [92]. A direct link between RSV and cell proliferation was also demonstrated in another study which showed how resveratrol induces reductions in cyclin D1, p38 MAP kinase, Akt, and Pak1 (p21 (RAC1) activated kinase 1) expression [76].

### 2.6. Hepatic Glucose Metabolism and Diabetes

Many studies have highlighted the positive influence of resveratrol on the liver of diabetic animals [93,94]. In fact, resveratrol can restore the liver’s regulatory role in glucose homeostasis [95,96]. This is associated with its ability to induce changes in the activities of carbohydrate metabolism enzymes in the livers of animals with diabetes. The molecular mechanisms behind this effect include decreasing the activity of key gluconeogenesis enzymes [97] and decreasing phosphoenolpyruvate carboxykinase protein levels, a rate-limiting enzyme for gluconeogenesis [98]. RSV also increases hexokinase and pyruvate kinase activity and decreases the activity of lactate dehydrogenase and glucose-6-phosphatase [99]. Furthermore, resveratrol has been shown to increase glycogen synthase, decrease glycogen phosphorylase, and increase the glycogen content in the liver [99]. All of these effects determine a shift in the metabolic pathways towards reduced hepatic glucose production. The effects of resveratrol in the liver are also accompanied by increases in insulin concentrations in the blood, which is, which is, at least in part, also responsible for these changes [100]. Therefore, the role of resveratrol in glucose metabolism is linked, first of all, to the increases in insulin levels with consequent reductions in hepatic glucose production, and secondly, to the increased use of glucose by insulin-sensitive tissues. The beneficial effects of resveratrol have also been linked to its direct and insulin-independent role in skeletal muscle and liver [101]. This property is accompanied by an antioxidative and anti-inflammatory effect of resveratrol and a further beneficial effect of resveratrol on metabolic disorders.

### 2.7. Chemical Liver Injury

Rivera et al. [102] examined the effect of resveratrol on CCI4-induced hepatotoxicity (carbon tetrachloride). After liver CCl4-induced damage, in cultured cells of hepatocytes, lipid peroxidation and γ-glutamyl transpeptidase activity are significantly increased [103]. In this context, resveratrol partially prevents increases in lipid peroxidation and γ-glutamyl transpeptidase [104]. Additionally, this effect was obtained at a dose of 50 mg/kg/day for 14 days in a model of chemically induced acute liver injury [105]. These hepatoprotective effects are associated with the antioxidant and cleansing properties of RSV, through a reduction in the activity of xanthine oxidase, a partial restoration of GSH, in addition to its ability to inhibit apoptosis [105,106]. Resveratrol has also been shown to be protective against heat stress-induced hepatic damage [107]. Therefore, the role of resveratrol in liver protection against chemical damage derives mainly from its ability to suppress oxidative stress and apoptosis.

### 2.8. Resveratrol and Liver Viruses

The ability of resveratrol to inhibit the replication of infective viral agents in Cytomegalovirus (CMV), varicella-zoster, influenza A and herpes simplex virus has been extensively investigated [108,109,110,111]. Nakamura et al. [112] found that resveratrol significantly influenced viral hepatitis C (HCV) RNA replication within the HuH-7 OR6-derived cell test system [112]. RSV additionally prevented the formation of hepatic steatosis in C57BL/6 mice infected with recombinant core HCV adenoviruses by inhibiting the expression of SIRT1 and PPAR-α (peroxisome proliferator-activated receptor-α) [113]. In transgenic hepatitis B virus (HBV) X protein (HBx) mice, RSV at a dose of 30 mg/kg/day had beneficial pleiotropic effects on intracellular reactive oxygen species, hepatocyte proliferation, and lipogenic genes. This resulted in delayed HBx-mediated hepatocellular carcinogenesis and significantly reduced the incidence of HCC [114,115]. In contrast, in another study, RSV activated hepatitis B virus (HBV) transcription, most likely due to its ability to stimulate SIRT proteins which activate HBV gene expression [114,115]. Therefore, the studies conducted so far have yielded contradicting evidence on the effects of resveratrol on HCV and HBV.

## 3. Resveratrol and Liver Disease: Scientific Evidence in Animal Models

### 3.1. Paracetamol and Resveratrol

The most common cause of acute liver failure worldwide is the use of paracetamol [116]. Paracetamol poisoning is a form of pharmaceutical intoxication stemming from high the administration or repeated administration of this over-the-counter analgesic [117]. Known also as acetaminophen, paracetamol is a known hepatotoxin, which induces liver damage mainly by oxidative stress [118,119]. Liver damage does not result from paracetamol itself, but from one of its metabolites, NAPQI (N-acetyl-p-benzoquinone imine). NAPQI reduces the concentration of glutathione, a natural antioxidant, in the liver and causes direct damage to liver cells leading to liver failure [120]. Risk factors for toxicity include chronic excessive alcohol intake, fasting or anorexia nervosa, and the use of certain medications such as isoniazid [121]. Thanks to its free radical scavenging properties and antioxidant effects, resveratrol has been shown to elicit many hepatoprotective effects against paracetamol-mediated toxicity. Treatment of hepatocytes with resveratrol, before and after paracetamol use, showed a decrease in AST (aspartate aminotransferase), ALT (alanine aminotransferase), TNF-α, MDA (malondialdehyde), and a reduction in neutrophil infiltration [122]. Other studies—also on mice—showed a reduction in ALT levels and interleukin 6 (IL-6) after acetaminophen-induced hepatic injury [123]. 

To better understand paracetamol-induced liver injury, two mouse models have been used. The two mouse strains, C57BL/6 and BALB/c are predominantly characterized by the development of Th1 and Th2 responses, respectively. After intraperitoneal administration of paracetamol, more severe liver damage was observed in C57BL/6 mice compared to BALB/c mice. By the same modality, after intraperitoneal paracetamol administration, hepatic mRNA expression of TNF-α and IL-6 was measured. TNF-α, a pro-inflammatory marker, was highly induced 24 h after paracetamol administration in C57BL/6 mice, while there was no change in BALB/c mice. IL-6 mRNA expression, an anti-inflammatory marker, was higher in the livers of BALB/c mice than those of C57BL/6 mice 24 h after administration. Furthermore, treatment of CD-1 mice- another sensitive strain- with resveratrol, protected the mice against acetaminophen-induced liver damage, and in mice, with attenuated toxicity, both lower TNF-α expression and higher IL-6 expression levels were found [123].

### 3.2. Ethanol and Resveratrol

The high production of alcohol-derived acetate triggers mechanisms that promote inflammation [124,125,126,127]. The metabolic activities of cytochromes on alcohol metabolites cause the release of free radicals, which, in turn, stimulate inflammatory processes in the liver [128,129,130]. 

Studies have shown the potential therapeutic utility of resveratrol in alcohol-induced liver damage. Bujanda et al. [131] demonstrated that resveratrol administered in drinking water prevents ethanol-induced liver injury, mortality, and oxidative stress in mice. The described effects are also associated with the decrease in ALT, AST, MDA, and the reversal of the increase in IL-1 and transaminase levels induced by ethanol. Chronic alcohol intoxication was induced by the progressive administration of up to 40% alcohol in drinking water. Mice given RSV received 10 mg/mL in drinking water and were assessed through transaminases, IL-1, and TNF-α blood levels. Histological evaluation of liver damage was performed and survival among animals was recorded. During the 10-week course of ethanol administration, a mortality of 100% was observed in the control group, but only 50% in the resveratrol group. Resveratrol alleviated alcohol-induced fatty liver disease in male mice by increasing the activity of AMPK and SIRT1, which are involved in the control of liver lipid metabolism pathways [28]. The hepatoprotective role of resveratrol is also linked to its antioxidant properties, this is evident from studies conducted by Kasdallah-Grissa et al. [132,133]. In these studies, dietary resveratrol prevented ethanol-induced lipid peroxidation in rats, resulting in a decrease in MDA (Malondialdehyde) content, an indicator of oxidative stress. For this purpose, recruited male Wistar rats were divided into three groups. The control group received a daily intraperitoneal injection of 0.9% saline. The second study group of rats was injected daily with 35% ethanol at 3 g/kg body weight. The third group was given the same dose of ethanol and supplemented with RSV (5 g/kg) in the standard diet. The treatment lasted 6 weeks and resulted in a 51.5% increase in MDA levels in the liver. However, in rats treated with ethanol in combination with RSV, the increase in MDA levels was significantly reduced, almost to the level observed in control rats. Resveratrol also produced an increase in antioxidant enzymes, namely SOD, catalase (CAT), and GPx.

### 3.3. Resveratrol in Chemical-Induced Hepatotoxicity

Many chemicals can cause liver damage. Resveratrol can positively contribute to this kind of chemical-induced damage. One of the hepatotoxic chemicals, cadmium, was investigated. Resveratrol in mice models treated with cadmium prevents oxidative damage and hepatotoxicity. Cadmium-induces hepatic lipid peroxidation (LP—expressed as malondialdehyde production) by decreasing GSH content and inhibiting catalase and GPx activity [134,135]. Pre-administration of RSV (20 mg/kg body weight, per os)/daily to male mice, for 3 days, followed by (7 mg/kg b.w., subcutaneous) cadmium chloride, compared to control animals, showed a reduction in glutathione (GSH), catalase (CAT), and glutathione peroxidase (GPx) levels in liver homogenates [134,135].

Another hepatotoxic chemical of interest is naphthalene. Naphthalene-induced liver damage and resveratrol hepatoprotective pharmacological effects have been investigated in mice. Treatment with resveratrol for 30 days reversed the increase in AST and ALT enzyme activity, as well as the cytokine levels of TNF-α, IL-1β, and IL-6, while improving total antioxidant capacity [136]. A single administration of resveratrol immediately following pyrogallol administration attenuated pyrogallol-induced hepatotoxicity and increased oxidative stress in mice. Resveratrol reduced AST and ALT levels, as well as the bilirubin concentration while increasing GSH content. Resveratrol also reduced the pyrogallol-induced increase in the activity of the metabolizing enzymes CYP1A2 (cytochrome P450 1A2) and CYP2E1 (cytochrome P450 2E1) [137]. A recent study by Farghali et al. [138] demonstrated that administration of resveratrol attenuated inflammation in d-galactosamine-sensitive and LPS-induced hepatitis [139]. This was highlighted by a reduction in TBARS (thiobarbituric acid reactive substances), AST, ALT, conjugated dienes, and bilirubin levels.

### 3.4. Resveratrol in Liver Damage Induced by Atherogenic Diet

High content atherogenic diets induce significant liver damage in both humans and laboratory animals and are responsible for fatty liver disease [140,141]. Studies have demonstrated the hepatoprotective effects of resveratrol in the high-fat dietary model of liver damage [142,143,144,145].

Ahn et al. [146] showed that resveratrol added to the diet of mice significantly reduced lipid, triglyceride, and cholesterol levels, while also suppressing the diet-induced high-fat expression of genes involved in lipid metabolism. The addition of resveratrol in high-fat diet-fed rats, after 8-weeks of treatment prevented lipid peroxidation, reduced TNF-α levels, and attenuated nitric oxide synthase expression, while increasing the activity of CAT, SOD, and GPx enzymes [23,147]. In another study using the same experimental model [148], resveratrol not only reduced cholesterol and lipid levels but also reduced mRNA expression of HMG-CoA (β-Hydroxy β-methylglutaryl-CoA) reductase [149]. In this study, resveratrol also increased the Apo A-l/Apo B ratio.

### 3.5. Resveratrol in Liver Ischemia-Reperfusion and Surgical Transplant 

The ischemia-reperfusion currently represents a critical event in terms of the outcome of liver transplantation [150]. Plin et al. [151] showed that resveratrol is protective against injury caused by ischemia-reperfusion in rats. The study evaluated the role of RSV in preventing 40 h cold storage-induced liver injury followed by hot reperfusion. Resveratrol offered protection and prevented oxidative stress and inflammation associated with ischemia-reperfusion in rats [152]. Treatment with resveratrol prevented lipid peroxidation, and enhanced SOD, CAT, and GPx activity, reducing aminotransferase levels, suppressing cytokine production, and improving vascular function [153,154]. The anti-inflammatory, antioxidant, and pro-apoptotic actions of resveratrol represent the basis of its positive effect on the transplanted rat liver (allograft). Resveratrol increased survival time in rats after liver transplantation and was also associated with decreased AST activity and decresed levels of cytokines IL-2 and interferon-γ (INF-γ). Resveratrol also inhibited signalling pathways, such as NF-κB, and attenuated the expression of important signalling intermediates, such as IβB kinase and protein kinase C Additionally, RSV also induced apoptosis in lymphocytes after liver allograft transplantation, as evidenced by an increase in the Bax/Bcl-2 (BCL2 associated X/B-cell lymphoma 2) ratio [155]. Kirimlioglu et al. [156] demonstrated the hepatoprotective and antioxidant properties of resveratrol in rats undergoing partial hepatectomy. The protective effect of resveratrol was associated with a reduction in lipid peroxidation and NO content and an increase in GSH content.

### 3.6. Resveratrol in the Irradiation-Induced Liver Injury Model

Velioğlu-Öğünç et al. [157] demonstrated the protective role of resveratrol in irradiation-induced liver damage. In this study, rats were treated with resveratrol (10 mg/kg/day) for ten days. They were then exposed to full-body radiation (800 cGy) and resveratrol treatment was continued for another 10 days after irradiation. Irradiation caused a significant decrease in glutathione levels and myeloperoxidase activity, while the collagen content increased in the liver and ileum tissues. Likewise, the plasma levels of lactate dehydrogenase and pro-inflammatory cytokines, 8-hydroxy-2’-deoxyguanosine, and apoptosis were elevated. Treatment with resveratrol reversed all of these biochemical indices, prevented lipid peroxidation in male rats undergoing X-ray irradiation, increased the GSH content, and reduced collagen levels. Its role in irradiation-induced liver damage is, therefore, essentially correlated to its antioxidant activity (Table 1).

## 4. Liver Disease and Resveratrol: Clinical Trials

### 4.1. Resveratrol and Non-Alcoholic Fatty Liver Disease

Non-alcoholic fatty liver disease (NAFLD) affects up to 30% of adults in Western countries and at least 15% in Asian countries. It is the most common form of liver disease worldwide and its prevalence is expected to increase in years to come [158]. NAFLD is characterized by the accumulation of excessive fat in the liver, mainly due to pathological processes such as triglycerides accumulation, steatosis, and non-alcoholic steatohepatitis (NASH) [159,160]. NAFLD and NASH can ultimately develop into cirrhosis and result in liver failure [161]. In the United States NAFLD is the third most common cause of liver transplant [162]. NAFLD falls under metabolic risk factors such as insulin resistance, DM (diabetes mellitus), hyperlipidaemia, cardiovascular diseases, and chronic kidney disease [163,164].

The clinical studies conducted so far have generally been too short to reveal noticeable impacts on liver structure. In fact, even in a 3-month study with 150 mg of resveratrol administrated daily to overweight or obese insulin-resistant patients with NAFLD, no effect was highlighted in terms of liver fat content or cardio-metabolic markers [165]. This study, although conducted using a large sample size did not take into consideration the importance of the dose-dependent effect of resveratrol due to its low bioavailability [166]. 

The dose of 150 mg may was probably too low. Several systematic meta-analyses have shown poor or no effects of resveratrol on the reduction in liver enzymes, anthropometric parameters, and/or blood chemistry values [167,168]. On the other hand, another analysis showed an interesting reduction in TNF-α in NAFLD patients [169]. A recent meta-analysis on 17 double-blind, randomized, placebo-controlled clinical trials showed beneficial effects [166], in particular, a significant reduction in blood pressure levels. This was highlighted by a meta-regression analysis of longer periods treatments, and inevitably, the highlighted results were supported by a dose-response relationship effect [170]. 

A clinical trial with a micronized formulation of trans-resveratrol in NAFLD patients highlighted promising results and therefore bodes well for the treatment of this condition. In fact, this better absorbable formulation exerted beneficial effects reducing liver fat, AST, ALT, GGT (gamma glutamyl transferase), and insulin resistance levels. Although the treatment duration, resveratrol dose, and formulation could have been the cause of these effects, the patient sample size was too small to confirm this [171]. This elucidates the conclusion that liver macro-modification requires long-term high-dose/good-bioavailability resveratrol treatment in NAFLD patients. Obviously, many more clinical studies are required to confirm this evidence.

### 4.2. Effect of Resveratrol on Diabetes Mellitus

The most important biological consequences of diabetes are mainly due to the disturbance in oxidative balance, strictly linked to the pathogenesis of several disease-related complications [172,173]. Resveratrol improves endothelial function, increases the oxidation of fatty acids in the liver, and decreases oxidative stress [174], leading to an improvement in insulin sensitivity [175]. A study in which 10 overweight subjects with impaired glucose tolerance were administered 1, 1.5, or 2 g of resveratrol daily for four weeks, participants showed an improvement in insulin sensitivity and postprandial glucose levels. In another clinical study, 17 volunteers with T2D (type 2 diabetes) were treated with 150 mg/day of RV for 30 days, showing a decreased intrahepatic lipid content and systolic blood pressure. Similarly, when overweight and obese men were exposed to RSV for two weeks, 1 g in the first week and 2 g in the second one, a reduction in intestinal and hepatic lipoprotein particle production was observed. RV decreased the production rate of ApoB-48 (apolipoprotein B-48) and both the production and fractional catabolic rates of ApoB-100 (apolipoprotein B-100), compared to a placebo [176,177]. Similar results, with no improvement in steatosis, insulin resistance, abdominal fat distribution, and plasma lipids have been highlighted in 20 overweight/obese men with non-alcoholic fatty liver disease after receiving 3 g/day RSV for eight weeks [35]. Some studies have analyzed the effect of resveratrol on obesity. A daily RSV dose of 150 mg/day administered for four weeks was given to 10 obese adults. Resveratrol supplementation suppressed postprandial glucagon, which may be important for the treatment of type II diabetes, as an excess of this hormone contributes to the patient’s hyperglycaemia [178]. 

Resveratrol was also found beneficial in type 1 diabetic patients. A two-month trial with 500 mg capsules of resveratrol, administered twice a day reported beneficial effects, such as a reduction in HbA1c (glycosylated haemoglobin), FBS (fasting blood sugar), and oxidative stress marker levels [179]. A recent meta-analysis even highlighted that in type 2 diabetic patients, resveratrol can reduce C-reactive protein (CRP) levels [180]. 

### 4.3. Cancer and Resveratrol

Thanks to their potent anti-inflammatory effect, natural compounds, such as resveratrol have gained significant attention for the treatment of many diseases, including cancer [62,181]. The insulin-like growth factor (IGF) signalling pathway, including IGF, IGF-binding proteins (IGFBP), and IGF receptors, is related to anticarcinogenic effects related to dietary restriction. In parallel, RSV can act as a preventive chemo-agent and caloric restriction mimetic in humans by reducing IGF-1 (insulin-like growth factor-1) and IGFBP-3 (insulin-like growth factor-binding protein 3) as seen in 40 healthy volunteers who consumed RSV at doses of 0.5, 1.0, 2.5, or 5.0 g per day for 29 days, leading to a reduced risk of cancer [182]. Moreover, there was a dose-dependent effect, as the largest reduction was seen at a dose of 2.5 g. Therefore, the preventive chemo action of RSV in humans was shown to act through the IGF system, which serves as a biomarker [183]. The only study available which has studied malignant hepatic tissue derived from patients with colorectal cancer, highlighted that 5 gr of micronized resveratrol administered for 14 days increased both the resveratrol and cleaved caspase-3 concentration in the malignant hepatic tissue with no safety issues [184].

### 4.4. Obesity and Resveratrol

In an 11-subject crossover study, RSV mimicked the effect of calorie restriction by reducing metabolic rate, activating AMPK in the muscle, and increasing levels of SIRT1 and peroxisome activity. It also increased citrate synthase activity and decreased the content of lipids within the liver, circulating glucose levels, triglycerides, alanine aminotransferase, and other inflammation markers. The evaluation index of the homeostatic model was also improved after the intervention [185]. To analyze the long-term effects of various polyphenols on the metabolic profile, RSV supplements and epigallocatechin-3-gallate (80 and 282 mg/day, respectively) were administered to 38 overweight or obese subjects over a period of 12 weeks. An increase in mitochondrial capacity and stimulation of fat oxidation was observed, along with an improved oxidative capacity of skeletal muscles and preservation of fasting and postprandial fat oxidation. Consequently, the concentration of triacylglycerol remained unchanged after RSV treatment, unlike in the placebo group, however, no improvement in insulin resistance was found in the peripheral, hepatic, or adipose tissues [186]. Unfortunately, a randomized, double-blind, and placebo-controlled clinical trial in overweight and insulin-resistant patients who received resveratrol at a dose of 75–150 mg/day for 12 weeks showed no effect on Liver fat content [165]. A recent meta-analysis of randomized controlled trials in patients with metabolic syndrome showed that resveratrol significantly reduced not only the total cholesterol levels, but also increased GGT, with an overall potential cardioprotective effect [187].

### 4.5. Effect of Resveratrol on Other Health Conditions Associated with Oxidative Stress and Inflammation

**Figure 1:**
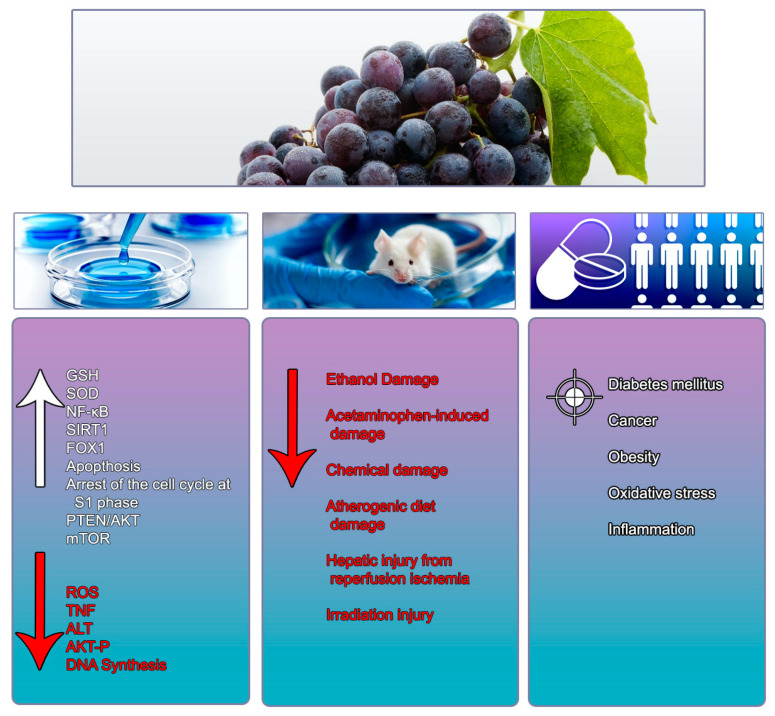
Summary of the main molecular mechanisms and physiological effects exerted by Resveratrol in vitro, in vivo and clinical studies. Red arrow (↓) indicates down-regulation; white arrow (↑) indicates up-regulation. GSH: glutathione; SOD: superoxide dismutase; NF-κB: nuclear factor kappa-light-chain-enhancer of activated B cells; SIRT1: sirtuin 1; FOX1: forkhead protein O box; S1:. Synthesis Phase; PTEN/AKT: phosphatase and tensin homolog/protein kinase B; mTOR: target of rapamycin in mammals; ROS: reactive oxygen species; TNF: tumor necrosis factor; ALT: alanine aminotransferase; AKT-P: protein kinase B.

The antioxidant effects of RSV have been extensively highlighted in the literature. In a study involving 10 healthy subjects, the administration of a single dose of 5 g RSV showed a significant increase in tumour necrosis factor-α (TNF-α) in patients’ plasma after 24 h. This increased production of TNF-α, as well as the inhibition of IL-10, was confirmed by analysis of peripheral blood mononuclear cells (PBMCs), which were activated through several toll receptor agonists [188]. In a separate trial, RSV’s effect on immune cells was evaluated in nine healthy men and women who ingested 1 g/day of RSV capsules for 28 days. The results showed that RSV induced an increase in circulating T cells and was consequently able to reduce the plasma levels of proinflammatory cytokines TNF-α and monocyte chemo-attractant protein 1. Moreover, RSV increased the antioxidant capacity of plasma with a consequent reduction in oxidative stress markers involved in DNA damage [189]. Administration of 75 mg/day of RSV every 12 weeks to postmenopausal women with normal weight and glucose tolerance, highlighted no changes in inflammatory markers, body composition, resting metabolism, plasma lipids, in the liver, in the skeleton density muscles mass, and adipose tissue or insulin sensitivity [190]. In conclusion, patients undergoing peritoneal dialysis in oral therapy with 450 mg of RSV delivered daily over 12 weeks, demonstrated improved urinary ultrafiltration and decreased vascular endothelial growth factor, fetal liver kinase-1, and angiopoietin- 2 (markers of angiogenesis) levels when ingested at the highest dose available [191]. A possible reason for the conflicting results could be that a low dose or a single high dose [188] has no positive effects on health and may even cause undesired effects. Results suggest that repeated and moderate administration of RSV (>450 mg) is better and safer than a single high dose [189,191] (Table 2 and Figure 1).

## 5. Conclusions, Future Perspectives and Limitations

Liver diseases remain a heavy health burden worldwide. This group of diseases requires new and safe therapeutic options for treatment. In this regard, resveratrol could be a promising option, as shown throughout our review. 

Resveratrol is already widely used as a dietary supplement. However, studies so far have not found specific doses and administration intervals for this compound. Possible therapeutic indications of RSV would largely benefit from a targeted approach. Understanding the correct use of this molecule in the different liver pathological settings could open new therapeutic scenarios. RSV could also benefit from association therapy with other drugs and compounds. Future perspectives are promising as RSV has been shown to have various beneficial effects in vitro, in vivo, and in clinical trials. An important limitation, in addition to RSV administration, is its bioavailability [184,192,193]. Although proven safe, RSV does not have optimal pharmacokinetics and pharmacodynamics properties [194,195,196]. 

Therefore, there is still much work to be done as the remaining knowledge gaps in relation to this compound are substantial. Well-designed clinical trials are necessary to improve the bioavailability of resveratrol and to understand its precise therapeutic usefulness, correct dosage, and treatment duration. 

## Figures and Tables

**Table 1:** Summary results of the intervention studies of Resveratrol in different models.

Improvement	Model	Dosage	Mechanism	Result	Reference
Steatosis	Rats	10 mg	Increase in SOD (superoxide dismutase), GPx (glutathione peroxidase), CAT (catalase) and a reduction in NOS (nitric oxide synthase) activity	Reduction in fatty acid deposition in liver	[23]
		[16]. 30 mg/kg [17]. 200 mg/kg	Activation of the AMPK/SIRT1 (AMP activated protein kinase/sirtuin 1) axis		[16,17]
Oxidative stress and inflammation		[36]. 500 mg od for six months	AMPK/SIRT1/Nrf2, ERK/p38, MAPK and PTEN/Akt signaling pathways, inducing autophagy through an mTOR-dependent or TFEB-dependent pathway	Synthesis of antioxidant molecules and the expression of related genes involved in the biogenesis of mitochondrial energy	[36,37]
		[43]. 10 to 1000 µM/L	Inhibition of the Nrf2 ubiquitination; promotion of the transcriptional functions of FoxOs	Induction of the transcription of antioxidant genes (CAT, SOD)	[41,42,43]
Liver fibrosis	Rats	[47]. 10 to 20 mg/kg od [50]. 25 to 150 µM/L od	Reduction in MDA (malondialdehyde) levels and increase in GPx and SOD levels, as well as the inhibition of mRNA expression of inflammatory mediators, including inducible NO, TNF-α (tumor necrosis factor-α), and IL-1β (interleukin-1β)	Reduction in portal pressure	[47,50,51]
		[52]. 20 mg/kg od for 3 weeks [54]. 20 mg/kg 3 days per week	Reduction in MDA levels and increase in GPx and SOD levels; inhibition of mRNA expression of inflammatory mediators (iNOS, TNF-α, IL-1β)	Reducing the liver tissue infiltration of inflammatory cells and fibrosis deposition	[52,53,54]
Cholestatic liver damage	Rat model of bile duct ligation		Reduction in TNF-α and IL-6 mRNA levels and in the number of CD68 (+) Kupffer cells recruited in the liver	Reduction in cholestatic damage	[55]
			Reduction in TIMP-1 and collagen Iα1 mRNA expression; increase of Ki67-positive hepatocytes	Reduction in fibrotic tissue deposition	[55]
Hepatocellular carcinoma and metastasis		[70]. Increasing doses of RSV, ranging from 4 to 32 µM/L [75]. 40 µM/L	Regulation of Fas and FasL levels; inhibition of the PI3K/Akt/mTOR pathway; inhibition of NF-κB	Induction of apoptosis	[70,71,72,77] [75,76,78] [79,80]
	Animal models exposed to carcinogenic chemicals (DENA)	[86]. 60 mg/kg od [88]. 50 mg/kg	Reduction in DENA-induced lipid peroxidation, iNOS; increase in protein carbonyl formation, Nrf2; reduction in the expression of hepatic TNF-α, IL-1β, and IL-6	Restoring of cellular antioxidant defenses Direct anti-inflammatory effect	[86,87,88]
		500 mg	Blocking of VEGFR expression through HIF-1a downregulation	Antiangiogenetic effect	[89]
		RSV dissolved in DMSO at a concentration of 25 mM	Reduction in cyclin D1, p38 MAP kinase, Akt, and Pak1 expression	Reduction in cellular proliferation	[76]
Hepatic glucose metabolism and diabetes		[98]. 6 mg od [99]. 5 mg/kg twice weekly for 30 days [100]. 10 mg/kg od	Decreased activity of lactate dehydrogenase, glucose-6-phosphatase, glycogen phosphorylase, and reduction in phosphoenolpyruvate carboxykinase protein levels; increases in hexokinase and pyruvate kinase activity, glycogen synthase.	Increased insulin metabolism	[97,98,99,100]
Chemical liver injury	Carbon tetrachloride-induced hepatotoxicity	[105]. 25/50/75/100 µM/L	Prevention of the increase in lipid peroxidation and γ-glutamyl transpeptidase activity	Antioxidant and antiapoptotic effect	[103,104,105,106]

Akt: protein kinase B, AMPK/SIRT1: AMP activated protein kinase/sirtuin 1, CAT: catalase, DENA: Diethyl nitrosamine, DMSO: Dimethyl sulfoxide, ERK: extracellular signal-regulated kinase, FoxO: forkhead protein O box, GPx: glutathione peroxidase, HFI 1: Hypoxia-inducible factor 1, IL-1β: interleukin-1β, MAPK: mitogen-activated protein kinase, MDA: malondialdehyde, NF-κB: nuclear factor kappa light chain enhancer of activated B cells, NO: nitric oxide, NOS: nitric oxide synthase, Nrf2: nuclear factor 2, PTEN: Phosphatase and tensin, RSV: resveratrol, SOD: superoxide dismutase; TFE: B transcription factor, TIMP: metallopeptidase inhibitor 1, TNF-α: tumor necrosis factor-α, VEGFR: vascular endothelial growth factor receptor.

**Table 2:** Summary results of the clinical trials of Resveratrol in different models.

Improvement	Dosage	Patients	Result	Reference
NAFLD	150 mg	Overweight or obese insulin-resistant patients with NAFLD	No effect on hepatic fat content	[165]
	50 mg Group 1 200 mg Group 2	44 patients, aged 29–70 years (*n* = 22 per group)	Reduction in the liver fat	[171]
Diabetes Mellitus	1 g, 1.5 g, or 2 g daily for 4 weeks	10 overweight subjects with impaired glucose tolerance	Improvement in insulin sensitivity and postprandial glucose levels	[175]
	150 mg/day for 30 days	17 volunteers with T2D (type 2 diabetes)	Reduction in intrahepatic lipid content and systolic blood pressure	[176,177]
	150 mg/day for 30 days	Evaluation of postprandial plasma responses of the incretin hormones and glucagon in 10 obese men	Suppression of postprandial glucagon	[178]
	500 mg capsules, twice daily for 60 days	13 patients with T1D (type 1 diabetes)	Reduction in HbA1c (glycosylated hemoglobin), FBS (fasting blood sugar) and oxidative stress markers	[179]
Cancer	0.5, 1.0, 2.5, or 5.0 g per day	40 healthy volunteers	Anticarcinogenica and caloric restriction mimetic effect by reducing IGF-1 and IGFBP-3	[182]
	Micronized RSV, 5 g daily for 14 days	Patients with colorectal cancer and hepatic metastases scheduled to undergo hepatectomy	Increase in cleaved caspase-3, a marker of apoptosis	[184]
Obesity	150 mg/day for 30 days	11 obese men in a randomized double-blind crossover study	In muscle: activation of AMPK, increased levels of SIRT1 and peroxisome activity. Increase in citrate synthase activity and decrease in lipid content within the liver, circulating glucose levels, triglycerides, alanine aminotransferase, and other inflammation markers	[185]
	EGCG (epigallocatechin-3-gallate) + RSV (282 and 80 mg/d, respectively) or placebo for 12 wk.	randomized double-blind study, 38 overweight and obese subjects	Increase in mitochondrial capacity and stimulated fat oxidation, without leading to increased tissue-specific insulin sensitivity	[186]
Health condition associated with oxidative stress and inflammation	5 g, single dose	Plasma cytokine levels were measured over 48 h after oral administration in 10 healthy subjects	Increased production of TNF-α; enhanced NF-κB activation	[188]
	1000 mg/day for 28 days	Repeated doses of resveratrol on circulating immune cells in healthy individuals	Increase in circulating γδ T cells and regulatory T cells Decrease in plasma levels of the proinflammatory cytokines TNF-α and MCP-1	[189]
	150 mg or 450 mg daily	72 patients undergoing peritoneal dialysis randomly assigned to 12 week treatment of low-dose or high-dose trans-resveratrol or a placebo	Improvement in mean net UF volume and UF rate through a reduction in VEGF, Flk-1 (fetal liver kinase-1) and Ang-2 (angiopoietin) levels	[191]

AMPK: AMP activated protein kinase, Ang-2: angiopoietin, FBS: fasting blood sugar, Flk-1: fetal liver kinase-1, HbA1c: glycosylated hemoglobin, IGF: insulin-like growth factor, IGFBP-3: insulin-like growth factor binding protin-3, MCP-1:monocyte chemoattractant protein-1, NAFLD: Non-alcoholic fatty liver disease, NF-κB: nuclear factor kappa light chain enhancer of activated B cells, RSV: resveratrol, SIRT1: sirtuin 1, T1D: type 1 diabetes, T2D: type 2 diabetes, TNF-α: tumor necrosis factor-α, UF: ultrafiltration, VEGFR: vascular endothelial growth factor receptor.

## Abbreviations

AC, acetyl; AIF, apoptosis-inducing factors; Akt, protein kinase B; AMPK, AMP activated protein kinase; ANIT, α-naphthyl isothiocyanate; APAF-1, apoptotic protease activating factor-1; ARE, antioxidant response element; ALT, alanine aminotransferase; AST, aspartate aminotransferase; Bax/Bcl-2, BCL2 Associated X/B-cell lymphoma 2; CAT, catalase; CRP, C-reactive protein; GSH, glutathione; CYP1A2, cytochrome P450 1A2; CYP2E1, cytochrome P450 2E1; DENA, Diethyl nitrosamine; DM, diabetes mellitus; DMN, dimethyl-nitrosamine; ERK, extracellular signal-regulated kinase; FA, fatty acid; FBS, fasting blood sugar; FoxO, forkhead protein O box; GGT, Gamma Glutamyl Transferase; GPx, glutathione peroxidase; GβL, G protein similar to β subunits; HbA1c, glycosylated haemoglobin; HBx, hepatitis B viral protein; HBV, hepatitis B virus; HCV, hepatitis C virus; HFD, high-fat diet; HIF-1α, hypoxia-inducible factor-1α; HMG-CoA, β-Hydroxy β-methylglutaryl-CoA; HO-1, heme oxygenase 1; IGF, insulin-like growth factor; IL-1β, interleukin-1β; INF-γ, interferon-γ; Keap1, Kelch-like ECH-associated protein 1; LKB1, hepatic kinase B1; MAFLD, metabolic associated fatty liver disease; MAP2K, mitogen-activated protein kinase kinase; MAPK, mitogen-activated protein kinase; MDA, malondialdehyde; MetS, metabolic syndrome; mSIN1, mammalian stress-activated protein kinase protein kinase 1; mTOR, target of rapamycin in mammals; mTORC2, mTOR Complex 2; NAD, nicotinamide adenine dinucleotide; NAFLD, non-alcoholic fatty liver disease; NAPQI, N-acetyl-p-benzoquinone imine; NF-κB, nuclear factor kappa light chain enhancer of activated B cells; NASH, non-alcoholic steatohepatitis; Nrf2, nuclear factor 2; NOS, nitric oxide synthase; P, phosphorylation; p53, phosphoprotein p53; PBMCs, peripheral blood mononuclear cells; PDK1, phosphoinositide-dependent kinase 1; PGC-1α, activator of gamma receptors activated by the proliferator of peroxisome 1α; PI3K, phosphatidylinositol 3-kinase; PIP2, phosphatidylinositol 4,5-bisphosphate; PIP3, phosphatidylinositol-3,4,5-triphosphate; PPAR-α, peroxisome proliferator-activated receptor-α; PTEN, Phosphatase and tensin homolog/protein kinase B; Rictor, mTOR’s rapamycin insensitive companion; SIRT1, sirtuin 1; α-SMA, Smac/DIABLO, second mitochondria-derived activator of caspases/direct IAP binding protein with low pI; α-smooth muscle actin; SOD, superoxide dismutase; STAT, signal transducer and transcription activator; TAG, triacylglycerols; TBARS, thiobarbituric acid reactive substances; TF, transcription factor; TFEB, EB transcription factor; TGF-β, transforming growth factor β; TIMP-1, tissue inhibitor of metalloproteinase-1; TNF-α, tumor necrosis factor-α; TRAIL, TNF-related apoptosis-inducing ligand receptors; TXA2, thromboxane A2; VEGF, vascular endothelial growth factor.
